# Divergent selection on locally adapted major histocompatibility complex immune genes experimentally proven in the field

**DOI:** 10.1111/j.1461-0248.2012.01791.x

**Published:** 2012-05-15

**Authors:** Christophe Eizaguirre, Tobias L Lenz, Martin Kalbe, Manfred Milinski

**Affiliations:** 1 Department of Evolutionary Ecology of Marine Fishes, GEOMAR| Helmholtz Center for Ocean ResearchKiel, 24105, Germany; 2 Department of Evolutionary Ecology, Max Planck Institute for Evolutionary BiologyPloen, 24306, Germany

**Keywords:** Adaptive radiation, host–parasite coevolution, local adaptation, major histocompatibility complex

## Abstract

Although crucial for the understanding of adaptive evolution, genetically resolved examples of local adaptation are rare. To maximize survival and reproduction in their local environment, hosts should resist their local parasites and pathogens. The major histocompatibility complex (MHC) with its key function in parasite resistance represents an ideal candidate to investigate parasite-mediated local adaptation. Using replicated field mesocosms, stocked with second-generation lab-bred three-spined stickleback hybrids of a lake and a river population, we show local adaptation of MHC genotypes to population-specific parasites, independently of the genetic background. Increased allele divergence of lake MHC genotypes allows lake fish to fight the broad range of lake parasites, whereas more specific river genotypes confer selective advantages against the less diverse river parasites. Hybrids with local MHC genotype gained more body weight and thus higher fitness than those with foreign MHC in either habitat, suggesting the evolutionary significance of locally adapted MHC genotypes.

## Introduction

The origin and extent of local adaptation among individuals in natural populations are a key aspect of adaptive evolution (Coyne & Orr 9; Hoekstra *et al*. 25). Locally adapted organisms are expected to have higher fitness in their local environment compared with migrants from adjacent populations (Kawecki & Ebert 31). Although the theoretical framework of local adaptation is well described (e.g. Gandon *et al*. 18; Nosil *et al*. 40; Bridle & Vines 6; Greischar & Koskella 19), the extent and the genetic basis for local adaptation in natural populations are still poorly understood (Hereford 24). This is due to the difficulty of identifying the exact selection pressure, the underlying mechanisms and the genes involved.

Biotic interactions such as prey–predator and host–parasites represent major selective factors in evolution (Haldane 20; Berryman 5). For parasites particularly, their geographic distribution can be very heterogeneous due to various ecological factors, like e.g. community structure, prevalence of intermediate hosts or habitat-specific transmission rates (Thompson 47; Kaltz & Shykoff 30). Contrasting environments are therefore likely to support distinct parasite communities, which would in turn require contrasting local immunogenetic adaptation of the hosts (Gandon *et al*. 18; Hedrick 22; Eizaguirre *et al*. 11).

The major histocompatibility complex (MHC) is a gene-dense region which encodes for cell-surface molecules involved in the recognition of parasite-specific antigens (Janeway *et al*. 26). Due to this essential function in the adaptive immune system, the evolution and the maintenance of the outstanding MHC polymorphism are thought to be driven by parasite-mediated selection (Milinski 38; Spurgin & Richardson 46; Eizaguirre *et al*. 13). Thus, the MHC represents a genetic basis for adaptation of vertebrate hosts to coevolving parasites. Combined with sexual selection, parasite-mediated selection acting on the MHC has the potential to drive the evolution of local adaptation and ultimately even speciation (Eizaguirre *et al*. 11).

Over the last decades, a growing body of research focused on understanding the mechanisms maintaining the MHC polymorphism. MHC-associated parasite resistance through heterozygote advantage, allele divergence and/or specific MHC allele–parasite associations has been proposed as potential mechanism (e.g. Oliver *et al*. 41; Wakeland *et al*. 48; Eizaguirre *et al*. 13; for reviews, see Bernatchez & Landry 3; Spurgin & Richardson 46). However, under homogeneous parasite selection, these mechanisms alone do not explain the observed large allelic diversity at the metapopulation level. Therefore, the idea of parasites exerting divergent selection on locally adapted MHC allele pools in heterogeneous environments has arisen, suggesting how this unparalleled genetic diversity is maintained (Hedrick 22; Eizaguirre & Lenz 10). Several recent field surveys investigating MHC variation at different geographical scales and in heterogeneous habitats proposed that contrasting parasite communities may shape MHC composition, e.g. in mammals (Oliver *et al*. 42), in birds (e.g. Ekblom *et al*. 14; Loiseau *et al*. 36) and in fish (e.g. Wegner *et al*. 49; Fraser & Neff 17; Eizaguirre *et al*. 12). Decisive experiments are still missing though (Spurgin & Richardson 46; Maan & Seehausen 37).

Testing local adaptation experimentally is challenging. A particular genotype may, for instance, perform better than others in all experimental laboratory conditions, because by chance it happens to be best adapted to the general laboratory set-up. Furthermore, it is practically impossible to simulate and control for all relevant environmental parameters in the laboratory, and thus, local adaptation to habitat differences not investigated in the experiment may confound the results. This is particularly true when dealing with parasitism because most host species are confronted to a multitude of parasites and pathogens in their natural habitat.

To test for MHC local adaptation and to avoid potential laboratory artifacts, we conducted a reciprocal common garden experiment in the field. For this purpose, the three-spined stickleback (*Gasterosteus aculeatus*) represents an exceptional model organism. This species is widely distributed in the northern hemisphere and shows rapid parallel adaptation to freshwater habitats (Colosimo *et al*. 8). Sticklebacks colonised lakes and rivers after the last glaciations and regularly segregate into lake and stream/river ecotypes (e.g. Reusch *et al*. 45; Hendry *et al*. 23; Berner *et al*. 4). Particularly in Northern Germany, these populations seem to experience practically no gene flow (Reusch *et al*. 45; Eizaguirre *et al*. 12), although both physical migration in nature and breeding in the laboratory are possible (Rauch *et al*. 44). It is also known that fish ecotypes are confronted with contrasting parasite communities (Kalbe *et al*. 28) and show little overlap in their MHC allele pools, whereas homogenizing selection seems to act within habitat type (Eizaguirre *et al*. 12). These characteristics led us to a specific breeding design, introgressing MHC genes by producing second-generation hybrid lines of lake and river fish with an MHC genotype of either river or lake origin, but a randomized genetic background. This enabled us to focus on the MHC genotype effect without confounding genetic background or non-genetic maternal effects (Lexer *et al*. 34; Rauch *et al*. 44). Using replicated mesocosms, we simultaneously and reciprocally exposed these second-generation hybrid fish, which had been bred parasite free, to the lake and the river environments where their grandparental generation had been caught respectively. After 10 months of exposure, the fish were individually dissected and screened for all individuals of all macroparasite species.

This experimental set-up had three main goals. First, we tested whether MHC genotypes are associated with different resistance/susceptibility patterns under different natural parasite exposures independently of the genetic background. Second, we tested whether varying resistance/susceptibility patterns may stem from host MHC adaptation to the local parasite fauna. Here, we expected fish of lake and river MHC genotypes to perform better in their respective habitats when facing the full natural range of local parasites. Third, we intended to perform a well-controlled experiment, highly replicated within each of two different habitats at the expense of having no replications at the habitat level. Therefore, our possible findings might serve as a proof of principal for the capacity of MHC to become locally adapted.

## Material and Methods

### Breeding design

**Figure 1:**
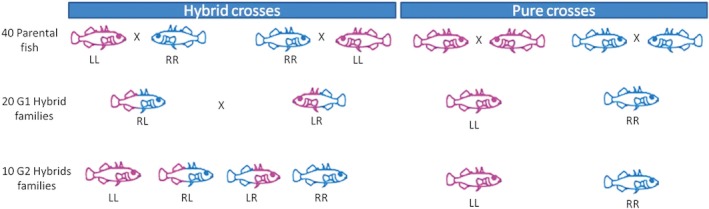
Experimental breeding scheme. Forty wild-caught fish were crossed with regard to their population of origin (20 from the lake, LL; 20 from the river, RR). The resulting 20 families (G1) carried mixed major histocompatibility complex (MHC) genotypes RL (*N* = 9 families) or LR (*N* = 11 families), with the first letter referring to the mother's origin. Inter G1 crosses were performed to obtain G2 hybrid families for which MHC genotypes segregate as follows: LL, RR, RL and LR. Each of the 10 G2 families was split into two groups: one was exposed to lake conditions, whereas the other was exposed to river conditions using two mesocosms each. Parallel pure second fish generations were obtained as control lines.

We sampled three-spined sticklebacks (*Gasterosteus aculeatus*) from two connected habitats, a small, slow-flowing river (Malenter Au, 54°12′16.19″ N, 10°33′32.93″ E, Germany) and a large lake (Großer Plöner See, 54°9′21.16″ N, 10°25′50.14″ E, Germany) in spring 2006. Like in other lake and river fish ecotypes in our study area, these populations exhibit reduced gene flow and show little overlap in terms of MHC alleles (Eizaguirre *et al*. 12). Fish were kept singly in 16-L tanks under spring conditions (12 °C, 12/24 light), with constant water flow and *ad libitum* food. After 6 weeks and transfer to summer condition (18 °C, 16/24 light), males were provided with artificial nesting material (sand and green nylon threads). For breeding, males and females were allowed to display natural behaviours and females spawned into the male's nest. Fish were randomly crossed with respect to their MHC genotypes, but not to their origin, to obtain lab-bred sibships with hybrid genomic background (Fig. 1). We obtained 20 hybrid G1 clutches. Eleven of these families had a mother of lake origin and nine had river mothers to control for maternal effects. The G1 generation was successively transferred to autumn, winter and spring conditions (4 months altogether), from when on the fish were kept singly. Finally, the fish were brought to summer conditions, where the males were provided with nesting material. Following the same protocol, we randomly intercrossed G1 hybrids. Since only one individual per G1 family was used for further breeding, all G2 families are genetically independent. Due to Mendelian segregation, in each of the 10 G2 hybrid families, four different MHC class II genotypes can be expected: RR, RL, LR and LL, with R and L referring to haplotype origin from river and lake habitat respectively. The first letter corresponds to the MHC haplotype inherited by the mother, whereas the second letter corresponds to the MHC haplotype inherited from the father. Importantly, MHC genotypes were determined at the end of the experiment. With original lake and river populations diverging strongly in their MHC allele pools, this design should result in G2 offspring with distinct river and lake MHC genotypes in a common randomised genomic background. Using G2 fish further eliminates potential ecotype- and MHC-related maternal effects. To control for potential unanticipated effects, five pure lake and five pure river G2 families were also obtained by intercrossing pure G1 families. The parental generation of those fish is the same as the one of the hybrid families.

### Field exposure

In autumn 2007 (at the age of 3.5 months), experimental fish were brought to the place in the lake and in the river where their grandparents had been caught respectively. Each of 38 mesocosms was stocked with 20 fish, partitioned as follows: 10 randomly picked fish from one of the 10 hybrid families and one fish from each of the five pure lake and five pure river families. For each hybrid family, we created four such replicates (except for one family where we achieved only two replicates). Two (one) mesocosms per family were placed into the lake and the two (one) others were placed into the river. The fish stayed in the mesocosms for 10 months from September 2007 to June 2008. In the middle of this period, one mesocosm was destroyed in the river by a thunderstorm. Once a week, all mesocosms were checked for dead fish.

The mesocosms were long and flat (1 m × 0.25 m × 0.6 m; length × height × width) to assure constant submergence even in the shallow river. Mesocosms consisted of stainless steel mesh and a stable frame. The 5-mm mesh allowed small particles and most invertebrates (food items and intermediate hosts of various parasite species) to pass through. We placed 19 mesocosms at an interval of 3 m in the centre of the river bed (about ca. 3 m wide and 1.5 m to 0.5 m deep). The other half of the set of mesocosms was placed at about 1–1.2 m depth at 2 m intervals in a usual stickleback breeding area in a shallow part of the lake (Kalbe *et al*. 29).

### Microsatellite typing

For each G2 fish, DNA extractions from a dorsal spine were performed using the Invisorb DNA Tissue HTS 96 Kit/R (Invitek, Berlin, Germany) on an automated platform (Tecan, Männedorf, Switzerland) following the manufacturer's protocol. All fish were typed for nine microsatellites combined in two different PCR multiplex protocols before and after the experiment (Kalbe *et al*. 29) to distinguish hybrids from pure rive/lake individuals. Information from microsatellites was also needed to identify fish before and after release in the mesocosms.

### MHC typing

Major histocompatibility complex class II*β* diversity was determined by reference strand conformation analysis (RSCA) according to Lenz *et al*. (33). We amplified the exon 2 of MHC class II*β* genes that encodes for the peptide-binding groove of the MHC molecule. MHC II*β* loci in the stickleback are duplicated and the number of sequence variants varies between individuals (Lenz *et al*. 33). This suggests the presence of paralogs and potentially a variable number of MHC II*β* loci in the populations. However, variants segregate in tightly linked haplotype blocks. With our breeding design, we were therefore able to track haplotype segregation and thus determine their lake or river origin respectively.

### Parasite load

As not all fish could be handled within 3 days as required for reliable parasite detection (Kalbe *et al*. 28, 29), dissections took place with regard to mesocosm replicates (80 fish) to assure that the same MHC genotypes are compared simultaneously between habitats of exposure. The dissections were spread over 4 weeks and the total number of fish recovered was 698. All external and internal macroparasites and ciliates were determined to the lowest taxonomic level possible using dissection microscopes (Kalbe *et al*. 28). Briefly, we recorded the abundance of parasites on the skin, on the gills, in the eyes, head kidney, body kidney, spleen, liver, gut, urinary bladder, swim bladder, gall bladder and muscles. The total parasite load of each fish was calculated as Shannon index. This allows summarising different combinations of parasites for each individual stickleback and to compare their total parasite burden quantitatively. The number of fish dissected per mesocosm ranged from 14 to 20 (mean ± SD, *u* = 18.83 ± 1.56).

### Statistical analysis

Statistical analyses were conducted using R statistical package (v. 2.12.2, R Development Core Team 43) and Primer v6 (Clarke & Gorley 7). All univariate tests are corrected for a possible gender effect. First, we investigated whether parasite communities varied between the two locations of exposure with an analysis of similarity (ANOSIM) on Bray–Curtis similarity matrix. Using a similarity percentage test (SIMPER), we identified the parasite contributing most to the difference in parasite community between habitats of exposure.

Then, we were interested in identifying the main source of variation observed in terms of parasite load (Shannon index). Therefore, we conducted a component variance analysis using a linear mixed-effect model with habitat of exposure, fish origin (pure line vs. hybrid cross), family, MHC origin and mesocosm ID as predictors.

To test whether MHC genotypes, independently of the genetic background, are associated with different resistance/susceptibility effects under different natural parasite exposures, we determined the effect of the interaction between all origins of MHC (lake LL, river RR or hybrids LR/RL genotypes) and the habitat of exposure on the parasite Shannon index in a linear mixed-effect model, where mesocosm ID and family were set as random factors.

In a following step, we tested whether any pattern of varying MHC genotype effects between habitat types could arise from local adaptation. To this end, we correlated MHC origin, this time only lake LL vs. river RR MHC genotypes to the Shannon parasite index and to the most contrasting parasite between both habitats of exposure (i.e. number of *Gyrodactylus* sp.) by performing two linear mixed-effect models as above.

As not only the presence of the different alleles is known but also their exact sequences have been obtained, we could investigate local adaptation at the sequence level. For this, the divergent allele advantage hypothesis (Wakeland *et al*. 48) provides a theoretical framework for adaptation. It suggests that genotypes with more divergent MHC alleles allow presenting a wider range of parasite-derived antigens to T cells and thus initiates an immune response against a broader spectrum of parasites. Because lake fish are confronted with a more diverse parasite community than river fish, we tested for the general resistance of lake MHC genotypes vs. the specificity of MHC resistance in river MHC genotypes. We calculated the MHC allele divergence (average pairwise amino acid p-distance between all MHC II*β* alleles of an individual) in our G2 fish. If our hypothesis is correct, there should be (1) a smaller allele divergence in river MHC genotypes than in lake MHC genotypes (tested using a nonparametric Wilcoxon test) and (2) a negative correlation between Shannon parasite index and average p-distance. To test for the latter one, we conducted a linear mixed-effect model with family and Cage ID set as random factors, the Shannon parasite index as dependent variable and individual allele divergence, MHC origin and habitat of exposure as predictors. To be conservative, we excluded fish with only one allele and thus a zero p-distance from the analyses which are more common in the river population and could artificially increase the experimental effect. However, results did not change when all the fish were included (data not shown).

Eventually, to relate MHC origin to a fitness proxy, we performed another linear mixed-effect model on the fish weight, accumulated during the 10 months of exposure, with *Gyrodactylus* load, MHC origin (LL vs. RR) and habitat of exposure as predictors. We included weight as residuals from a linear model with sex as predictor. Here as well, cage ID and family were set as random factors. For all mixed-effect models, we used the Restricted Maximum Likelihood for slightly unbalanced designs – due to some low mortality of fish.

### Extension of results

Conducting our translocation experiment with additional river/lake pairs would help identifying whether fish adapt to their local environment (i.e. population) or whether they adapt to the river/lake conditions more broadly. As each such replication of a river/lake pair is logistically extremely demanding and would necessitate over 3 years, we collected fish from an additional lake/river pair [Vierer See (Lake, *N* = 29, 54º7′31.94″ N, 10º 26′39.55″ E)/Schwale (River, *N* = 24, 54º0′24.47″ N, 10º9′2.04″ E)] and investigated whether signs of genomic signatures, i.e. high genetic distance in lake fish and lower in river fish, could be identified. Fish were part of another study (Eizaguirre *et al*. 12). As the genetic p-distance was not normally distributed, pairwise comparisons with Wilcoxon tests were performed.

## Results

### Contrasting MHC allele pools between populations

In the parental generation, we detected 22 different MHC II*β* alleles, distributed over 16 haplotypes in which the number of alleles varied from 1 to 3, suggesting a variable number of loci (see Lenz *et al*. 33 for accession numbers). One allele occurred in two different haplotypes – suggesting naturally occurring recombination. The ANOSIM confirmed that the two populations differed in their pools of MHC haplotypes (*R* = 0.47, *P* < 0.001, see also Eizaguirre *et al*. 12).

In all G2 hybrid lines but one, we could clearly determine the population origin of the different MHC haplotypes. This one family was excluded from all analyses due to overlapping parental genotypes. In the following, results focusing only on hybrid lines are thus obtained from nine hybrid families, carrying in total 22 different alleles, distributed over 13 haplotypes.

### Contrasting parasite communities between habitats

To confirm contrasting selection pressures between habitats of exposure, we investigated the macroparasite community in each habitat. Dissecting the second-generation (G2) hybrid fish after 10 months of exposure, we recorded a total of 24 different parasite taxa (Supplementary Table S1). A total of 22 different parasite taxa were found in fish exposed in the lake, whereas 14 different parasite taxa were counted in fish exposed in the river. Among these parasite species, 10 were lake specific, whilst only two were found in fish exposed to river conditions (Supplementary Table S1).

Parasite communities found in the fish differed between habitats of exposure (ANOSIM, *R* = 0.625, *P* < 0.001). A similarity percentage analysis of species contribution revealed that the average dissimilarity between parasites communities was 50.60%. The difference was mainly driven by the intensity of the monogenean *Gyrodactylus* sp. (25.31%) which was predominating in the river.

## MHC adaptation to local parasite communities

### Component variance analysis

Identifying the relative role of MHC genes in parasite resistance against the randomised genetic background was one of our main goals. First, we found that fish from the hybrid lines harboured intermediate parasite load compared with pure lines (Supplementary Figure S1). On the contrary, parasite load, estimated as Shannon index, was mainly a function of habitat type, explaining almost 38% of the observed variance. Whether fish were obtained from a pure line or a hybrid line explained about 20% of the observed infection variance. Eventually, MHC origin alone explained ∼ 10% of the observed variance which corresponds to about two-thirds of the variance explained by family variation (see Supplementary Table S2).

### MHC and population-specific parasite resistance

**Figure 2:**
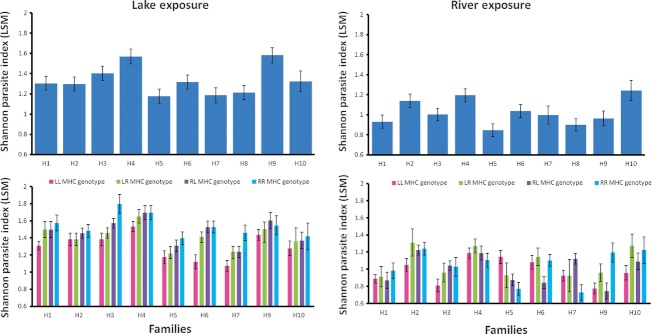
Effect of family and major histocompatibility complex (MHC) genotype on parasite load. The upper panels a & b show mean parasite load (Shannon index) of sticklebacks in the ten hybrid families, exposed to the lake and river habitat. The lower panels c & d show the parasite load for the four different MHC genotype origins in the nine hybrid families for which MHC origin could be determined. Bars show least square means (± standard errors, Table 1a).

First, if, independently of the genetic background, MHC effects are associated with local parasites, we should observe a significant interaction between the origin of MHC II*β* genotypes and the habitat of exposure on the contrasting Shannon parasite indices between lake and river. When testing the MHC effect against the variation across families (Fig. 2a and b), our hypothesis was supported by a significant interaction between MHC origin and habitat of exposure (*F*_3,607_ = 9.52, *P* < 0.001. Table 1a; Supplementary Table S3a for model estimates, Fig. 2c and d).

**Table 1:** Statistical details for two different linear mixed-effect models on (a) Shannon parasite index (total parasite load) for all four Major histocompatibility complex (MHC) genotypes, (b) Shannon parasite index only for LL and RR genotypes and (c) the specialist *Gyrodactylus* sp. (number of infecting Gyrodactylus parasites) with only LL and RR MHC genotypes

	(a) All MHC genotypes Shannon			(b) LL vs. RR Shannon			(c) LL vs. RR *Gyrodactylus* sp.		
			
	d.f.	*F*	*P*	d.f.	*F*	*P*	d.f.	*F*	*P*
Habitat of exposure	1,40	49.435	**< 0.001**	1,25	11.3443	**0.0024**	1,25	13.4608	**0.0012**
MHC origin	3,422	2.557	0.055	1,130	4.281	**0.041**	1,130	4.3286	**0.0394**
Habitat × MHC origin	3,607	9.521	**< 0.001**	1,133	1.4976	0.2232	1,133	1.4622	0.2287

Model parameter estimates are given in Supplementary Table S3a, b and c. Significant values (p < 0.05) are in bold.

**Figure 3:**
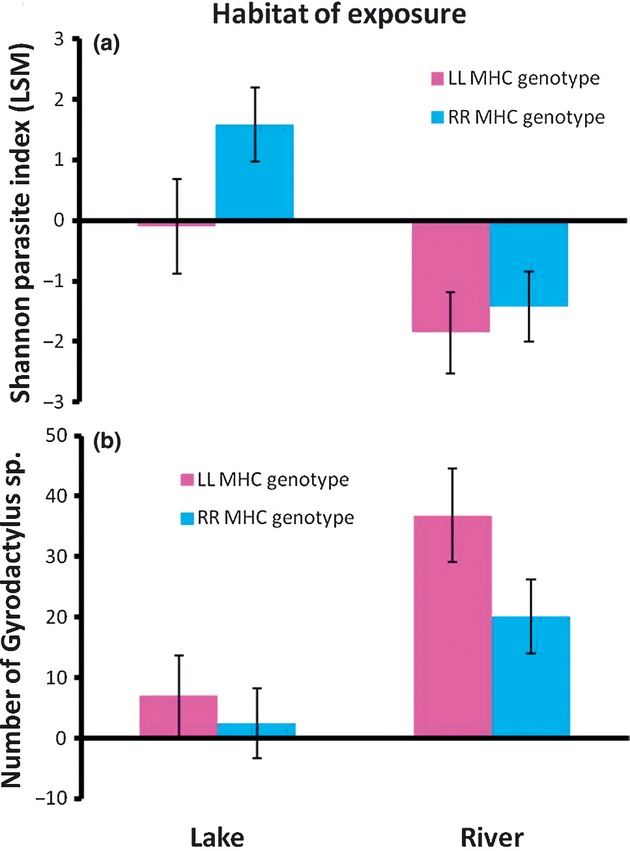
Local adaptation of major histocompatibility complex (MHC) origin. Parasite load of only the G2 hybrid sticklebacks carrying MHC genotype of different origin (LL vs. RR). (a) For the Shannon index including all parasites, (b) for the specialist *Gyrodactylus* sp. Bars show least square means of an independent linear mixed-effect model (± standard errors, Table 1b and 1c).

Second, if this pattern arises from MHC local adaptation, individual fish should do better in the habitat type where their MHC haplotypes coevolved with the local parasites. When comparing only LL ‘lake MHC genotypes’ (i.e. second-generation hybrids with lake MHC genotype but mixed genetic background) and RR ‘river MHC genotypes’ (i.e. second-generation hybrids with river MHC genotype but mixed genetic background), we found that lake MHC genotypes were associated with higher resistance than river MHC genotypes in both habitat types when considering the total parasite load (*F*_1,130_ = 4.28, *P* = 0.041, Supplementary Table S3b for model estimates, Fig. 3a). This result holds also true when *Gyrodactylus* sp. is excluded from the total parasite load (Supplementary Figure S2). Noteworthy, mixed MHC genotypes (RL and LR) harboured on average an intermediate parasite load compared to LL and RR MHC genotypes (Supplementary Figure S3).

As the two habitats of exposure differed substantially in their parasite communities, the overall parasite diversity (Shannon index) alone may not adequately represent all aspects of local parasite selection. The contribution of individual parasite taxa to the difference between habitats varied substantially as shown by the SIMPER test. The parasite with the highest contribution, *Gyrodactylus* sp., explained alone more than a quarter of the total difference between parasite communities. We found that *Gyrodactylus* sp. was much more abundant in river (*F*_1,25_ = 13.46, *P* = 0.001, Table 1c, see Supplementary Table S3c for model estimates), and fish carrying river MHC genotypes were indeed more resistant to this parasite, independently of the habitat of exposure (*F*_1,130_ = 4.33, *P* = 0.039, Fig. 3b; Table 1c). From these results, we hypothesised that lake MHC genotypes are more adapted to the broad spectrum of parasites present in the lake, whereas river MHC genotypes, due to the overall much lower parasite diversity in the river, are more targeted and adapted to fighting the usually most dominant parasite which consistently dominates river parasite communities over years (Eizaguirre *et al*. 12; Martin Kalbe, Mathias K. Wegner, Christophe Eizaguirre, unpublished data). Adaptation to different parasite diversities should be reflected at the MHC divergence level.

### General resistance vs. specific resistance against local parasites

Supporting our hypothesis, river MHC genotypes displayed lower sequence divergence than lake MHC genotypes (Wilcoxon test, *W* = 3178, *P* < 0.001, median_LL_ = 0.2465, median_RR_ = 0.2328). Also, as predicted by the allele divergence hypothesis, we found a significant negative correlation between Shannon parasite index and allele divergence after correction for possible confounding factors of number of MHC alleles and MHC origin (*F*_1,271_ = 5.61, *P* = 0.0185. Table 2a, Supplementary Table S4). No such correlation could be found for *Gyrodactylus* sp. alone (data not shown).

**Table 2:** (a) Summary of the linear mixed-effect model conducted on the Shannon parasite index with individual average amino acid p-distance between alleles [corrected for the number of Major histocompatibility complex (MHC) alleles] and habitat of exposure as predictors. (b) Summary of the linear mixed-effect model conducted on the fish weight with *Gyrodactylus* load, MHC origin and habitat of exposure as predictors. Weight is expressed as residuals from a linear model with sex as predictor

	d.f.	*F*	*P*
(a) Shannon parasite index			
Average p-distance	1,271	**5.6130**	**0.0185**
MHC origin	3,255	0.5166	0.6712
Habitat of exposure	1,25	**40.4518**	**< 0.0001**
Average p-distance × MHC origin	3,243	2.6102	0.0521
MHC origin × Habitat of exposure	3,269	1.3267	0.2660
(b) Fish weight			
Gyrodactylus load	1,87	1.5610	0.2149
Habitat of exposure	1,8	17.2869	**0.0030**
MHC origin	1,140	0.2090	0.6482
Gyrodactylus × MHC origin	1,81	4.8635	**0.0303**

Mesocosm ID and family were set as random factors. Significant values (p < 0.05) are in bold.

## Fitness proxy for local MHC adaptation

As the fish with lake MHC genotypes were overall less infected by the small array of parasites in the river, it remained to be tested whether the specific resistance of river MHC against the highly abundant river parasite *Gyrodactylus* sp. is evolutionarily significant, i.e. provides a net advantage to river fish. For this, we used fish weight, accumulated during the 10 months of exposure, as a fitness proxy. The interaction seen between MHC origin and *Gyrodactylus* sp. load on fish weight supports a net cost of *Gyrodactylus* sp. infection on fish carrying lake MHC genotypes in river habitat (*F*_1,81_ = 4.86, *P* = 0.0303, Table 2b, Supplementary Figure S4). Thus, hybrid fish with river MHC genotypes achieved a higher weight than those with lake MHC genotypes under high *Gyrodactylus* sp. exposure, which occurs mainly under river conditions.

## Extension of results

**Figure 4:**
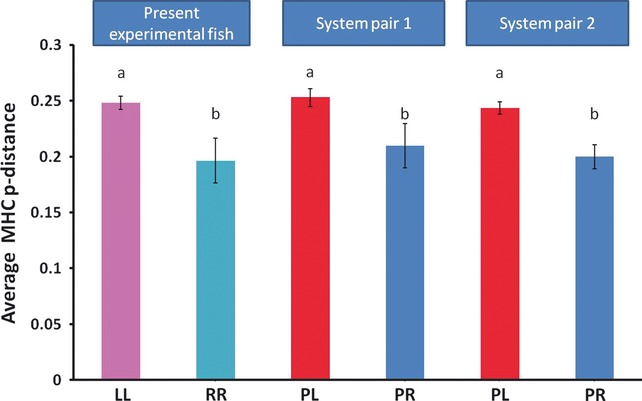
Major histocompatibility complex (MHC) divergence (p-distance) of the present experimental fish, the fish caught in the same population as the parents of the experimental fish (system pair 1, ‘Malenter Au’ river and the ‘Großer Plöner See’ lake) and fish captured in another lake/river system (system pair 2, ‘Schwale’ river, ‘Vierer See’ lake). MHC alleles and genotypes have been described in Eizaguirre *et al*. (12). Red depicts average MHC p-distance of fish caught in lake; blue is used for fish caught in river. Purple and light blue are used to depict genetic divergence observed in the present experimental fish. Bars connected by different letters are significantly different (pairwise test, *P* < 0.001).

To potentially generalize our findings, which suffer from a lack of replication at the habitat type level, an additional lake/river population pair from a previous study (Eizaguirre *et al*. 12) with comparable parasite communities was independently tested for population differences in MHC divergence. We found that fish with river MHC genotypes (whether hybrid RR from the translocation or pure RR from the natural populations) harbour MHC genotypes with lower MHC divergence than fish carrying LL or pure lake MHC genotypes (All pairwise wilcoxon tests between lake and river *P* < 0.001, Fig. 4).

## Discussion

Identifying a genetic basis for local adaptation is of prime interest in evolutionary biology (Hoekstra *et al*. 25; Barrett *et al*. 2). In heterogeneous host–parasite systems, theory predicts hosts and parasites to adapt to their local environment (Lively & Dybdahl 35; Hedrick 22). The characterisation of the extremely polymorphic MHC genes, which allow for host immunogenetic adaptation to a specific parasite-derived antigen repertoire (Eizaguirre *et al*. 13), enables us to investigate local adaptation in vertebrates (Bernatchez & Landry 3; Maan & Seehausen 37). Divergent locally adapted MHC allele pools should arise from MHC alleles being positively selected in some environments but negatively selected or neutrally evolving in others. Despite an increased interest in this type of MHC evolution, experimental work demonstrating MHC local adaptation is elusive (Rauch *et al*. 44; de Eyto *et al*. 16; Evans *et al*. 15; Spurgin & Richardson 46). This is likely due to the effort associated with identifying the wide array of parasites in a natural host population (Thompson 47) and the difficulty of disentangling the role of MHC genotypes from the genetic background (Rauch *et al*. 44).

In the present experiment, we exposed second-generation hybrid three-spined sticklebacks that segregated for MHC genotype origin (lake vs. river) but exhibited a common randomised genetic background to the natural lake and river habitat of their grandparents. With this design, we were able to estimate the relative contribution of the genetic background and MHC origin to parasite resistance, where habitat of exposure was the main cause of variance in parasite load (∼ 40%). Whereas the genetic background, here represented by pure and hybrid lines, explained about 20% of the observed variance in parasite load, another 10% could be attributed solely to the origin of MHC II*β* genotypes.

Dissecting this pure MHC effect further by looking at our experimental hybrid fish, we present evidence for an interaction effect between MHC genotypes and habitat/site of exposure on parasite load. The randomised genetic background of our experimental fish verifies that the effect is MHC specific and that this resistance/susceptibility effect is not universal but depends on the selecting parasites. Hence, parasite-mediated natural selection can play a crucial role in sorting MHC allele pools across heterogeneous habitats. Our results go beyond a previous attempt of determining the role of MHC genes in parasite resistance where no effect could be found (Rauch *et al*. 44). The stronger and significant effect in our experiment probably arose from the substantially extended time of exposure, which covered almost the entire lifespan of sticklebacks in those populations. This exposure duration allows for meeting sequentially occurring parasites and also seems important for some pathways of the adaptive immune system, where mounting an adaptive immune response takes up to 2 weeks in mammals and up to many weeks in ectotherms such as fish (Janeway *et al*. 26). Interestingly, our experiment revealed a more complex scenario of local adaptation than expected a priori. MHC genotypes were not adapted to their respective location, but instead to site-specific characteristics of the local parasite community: fish carrying lake MHC genotypes had a lower total parasite load, whereas fish carrying river MHC genotypes were less infected with the specialist parasite *Gyrodactylus* sp., which consistently dominates the river parasite community over years (Eizaguirre *et al*. 12). In both cases, this effect was qualitatively almost independent of the habitat of exposure. Lake sticklebacks do indeed harbour a higher number of and more diverse parasites (Kalbe *et al*. 28; Eizaguirre *et al*. 12), and this was again confirmed in our experiment. We hypothesised that coevolution with such a broad parasite community should drive the evolution of higher sequence divergence between alleles in lake MHC genotypes. Conversely, as river fish are exposed to a smaller range of parasites that is furthermore dominated by one abundant specialist parasite, they should have evolved a lower MHC allele divergence, but should have coevolved more tightly with the constantly most predominant parasite. Consistent with this idea, we found that lake MHC genotypes contained genetically more divergent alleles than river MHC genotypes. Correlating individual MHC allele divergence to parasite load, we found that the higher the allele divergence, the lower the parasite load. This is the first experimental demonstration of the divergent allele advantage, a refined idea of overdominance, where higher genetic diversity at the sequence level has been suggested to allow broader parasite recognition (Wakeland *et al*. 48; Lenz 32).

The lower body weight of fish with lake MHC genotypes in the river habitat (which are more heavily infected with *Gyrodactylus* sp.) suggests a net particular cost of *Gyrodactylus* sp. infection intensity, as already suggested in previous studies (e.g. Fraser & Neff 17). Reduced weight indices have regularly been associated with lower Darwinian fitness in fish and in sticklebacks, in particular (Kalbe *et al*. 29; Wootton 50). Such lower vigor would surely restrain lake fish with their lake MHC genotypes from reproducing in the river. Vice versa, the same applies to river fish whose river MHC is not adapted to the more diverse parasite community in the lake that is lacking a predominating parasite but instead harbours a broader range of parasites with potentially more equivalent fitness costs. This reciprocal local adaptation is likely to limit gene flow between both fish ecotypes and increases population divergence. Combined with female mate choice for specific MHC combinations and MHC resistance alleles (Aeschlimann *et al*. 1 Milinski 38), immunogenetic local adaptation could ultimately even lead to speciation (Eizaguirre *et al*. 11).

It is also worth noting that in our experiment, fish with hybrid MHC genotypes (one haplotype from each population source) displayed intermediate levels of parasite load. This suggests Mendelian inheritance for the observed resistance and thus supports the idea that resistance is determined by only a low number of genes in linkage disequilibrium or a gene complex such as the MHC. We cannot completely rule out the possibility that the observed effect is based on non-MHC genes in linkage disequilibrium with the MHC. However, *e*ach of the population-specific MHC haplotypes in our experimental families originated from several randomly wild-caught and unrelated parents of the given population, which reduces linkage disequilibrium between MHC and other genes around the MHC due to natural recombination in previous generations. The observed effect of sequence divergence at the MHC genes on resistance provides additional evidence that the observed effect can be related to the MHC, as sequence divergence at the MHC is unlikely to be informative for any other locus**.**

Overall, our results show experimentally that MHC genes provide the potential for local host adaptation to contrasting parasite communities, independently of the host's genetic background. In a next step, it remains to be investigated whether local adaptation of MHC genes is population specific or rather habitat type specific. The evolution of locally adapted MHC allele pools by means of parasite-mediated diversifying selection may explain the patterns found in numerous field surveys investigating MHC variation across populations in heterogeneous environments and contributes to the maintenance of the outstanding MHC polymorphism at the meta-population level.

## SUPPORTING INFORMATION

As a service to our authors and readers, this journal provides supporting information supplied by the authors. Such materials are peer-reviewed and may be re-organized for online delivery, but are not copy-edited or typeset. Technical support issues arising from supporting information (other than missing files) should be addressed to the authors.
