# Supplementary material for: Divergent selection on locally adapted major histocompatibility complex immune genes experimentally proven in the field
Source: Ecol Lett. 2012 May 15;15(7):723–31. doi: 10.1111/j.1461-0248.2012.01791.x (PMC3440595; doi:10.1111/j.1461-0248.2012.01791.x)

**Supplementary Information**

**Supplementary figure 1:** Shannon parasite index as a function of fish origin: PL stands for pure lake, PR for pure river and H for hybrids (All MHC origin pooled). *p<0.05, **p<0.001


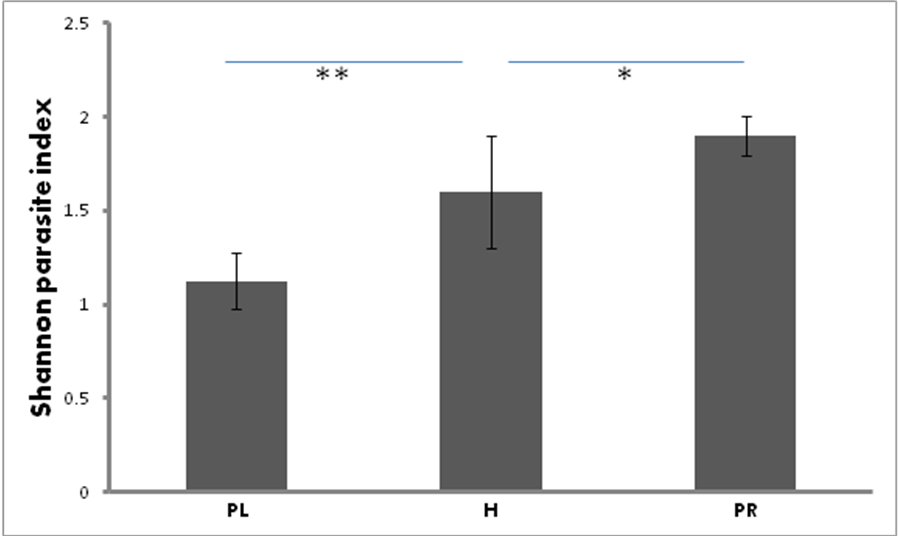

Supplement: Supplementary file 1 [file ele0015-0723-SD1.doc]
