# Supplementary material for: Divergent selection on locally adapted major histocompatibility complex immune genes experimentally proven in the field
Source: Ecol Lett. 2012 May 15;15(7):723–31. doi: 10.1111/j.1461-0248.2012.01791.x (PMC3440595; doi:10.1111/j.1461-0248.2012.01791.x)

**Supplementary figure 2:** Shannon parasite index without Gyrodactylus sp. as a function of fish MHC genotypes in hybrid lines: LL stands for lake MHC genotypes while RR stands for river MHC genotypes. Bars show least square means (+/- standard error).


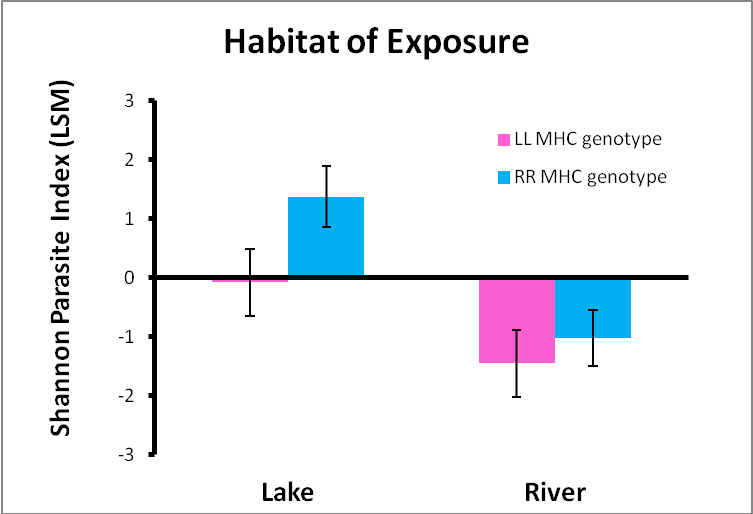

Supplement: Supplementary file 2 [file ele0015-0723-SD2.doc]
