# Supplementary material for: Divergent selection on locally adapted major histocompatibility complex immune genes experimentally proven in the field
Source: Ecol Lett. 2012 May 15;15(7):723–31. doi: 10.1111/j.1461-0248.2012.01791.x (PMC3440595; doi:10.1111/j.1461-0248.2012.01791.x)

**Supplementary figure 3:** Parasite load of G2 hybrid sticklebacks carrying MHC genotype of different origin (LL, LR, RL and. RR). a) for the Shannon index including all parasites, b) the specialist *Gyrodactylus sp.*. Bars show least square means (+/- standard errors).


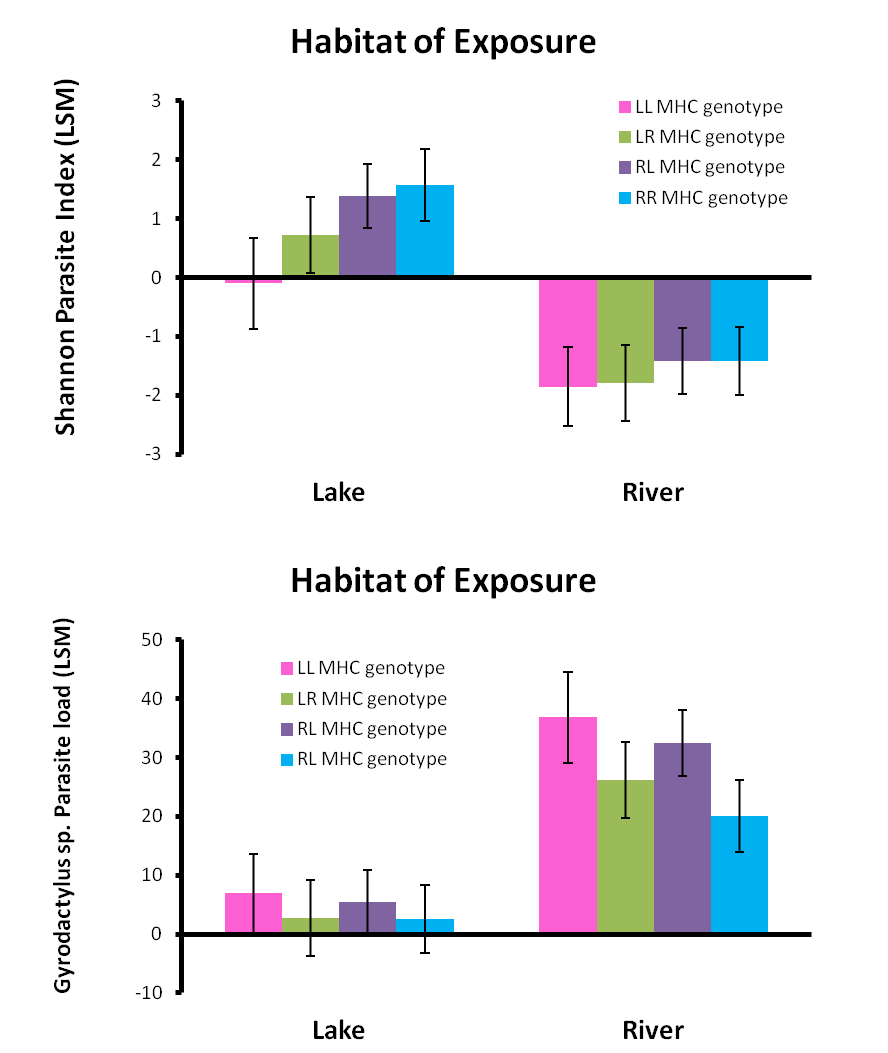

Supplement: Supplementary file 3 [file ele0015-0723-SD3.doc]
