# Supplementary material for: Divergent selection on locally adapted major histocompatibility complex immune genes experimentally proven in the field
Source: Ecol Lett. 2012 May 15;15(7):723–31. doi: 10.1111/j.1461-0248.2012.01791.x (PMC3440595; doi:10.1111/j.1461-0248.2012.01791.x)

**Supplementary figure 4:** Fish weight expressed as fitness proxy in relation to the number of Gyrodactylus sp. parasites recorded. Fish are split for LL and RR MHC genotypes. Outliers have been removed from the graphical representation. Regression lines are shown.


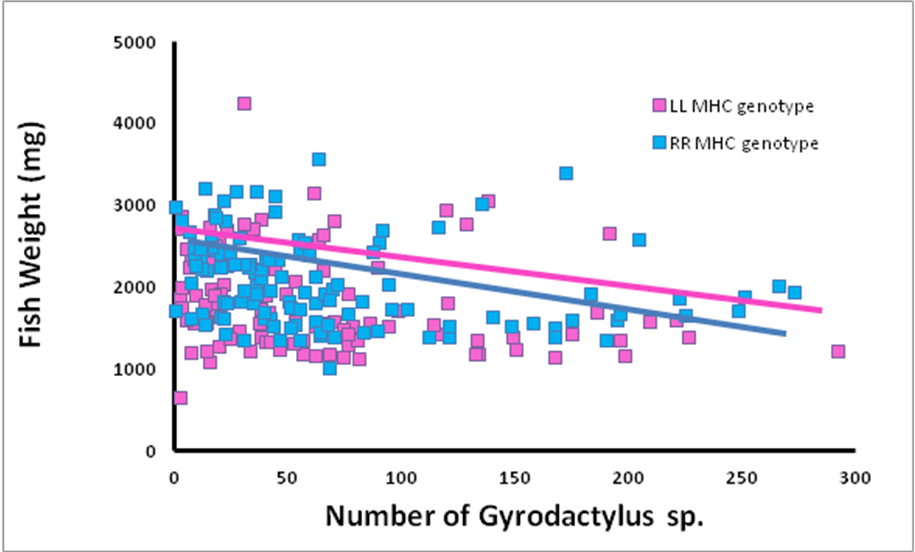

Supplement: Supplementary file 4 [file ele0015-0723-SD4.doc]
