# Supplementary material for: Divergent selection on locally adapted major histocompatibility complex immune genes experimentally proven in the field
Source: Ecol Lett. 2012 May 15;15(7):723–31. doi: 10.1111/j.1461-0248.2012.01791.x (PMC3440595; doi:10.1111/j.1461-0248.2012.01791.x)
Supplement: Supplementary file 5 [file ele0015-0723-SD5.doc]

**Supplementary Table 1:** Table summarizing the prevalence (percentage of infected hosts), the mean intensity (number of parasite individuals of a given species per infected host), and the maximum number of parasite of each recorded parasite in the 698 dissected fish. References are found below.

|  |  | **Lake exposure** | | | | **River exposure** | | | |
| --- | --- | --- | --- | --- | --- | --- | --- | --- | --- |
| Parasite species | Parasite strategy | Mean | Max | Median | Prevalence | Mean | Max | Median | Prevalence |
| Diplostomum sp. | Generalist^1^ | 18.04 | 68 | 16 | 99.18 | 16.88 | 75 | 14 | 97.14 |
| Apatemon cobitis | Generalist^2^ | 9.22 | 296 | 4 | 86.30 | 0.23 | 6 | 0 | 11.14 |
| Trichodina sp. | Generalist^3^ | 9.09 | 100 | 1 | 83.01 | 34.43 | 100 | 10 | 95.43 |
| Glochidia | Generalist^4^ | 5.53 | 92 | 3 | 76.71 | 0.06 | 14 | 0 | 1.14 |
| Gyrodactylus sp. | Specialist^5^ | 12.84 | 248 | 3 | 72.33 | 81.06 | 1073 | 46 | 99.43 |
| Apiosoma sp | Generalist^3^ | 4.64 | 100 | 1 | 58.63 | 33.86 | 100 | 10 | 69.14 |
| Cyathocotyle prussic | Generalist^6^ | 1.20 | 37 | 0 | 44.38 | 4.13 | 21 | 3 | 85.71 |
| Echinochasmus sp. | Generalist^7^ | 0.73 | 7 | 0 | 40.55 | 0.02 | 2 | 0 | 1.14 |
| Argulus foliaceus | Generalist^8^ | 0.84 | 16 | 0 | 38.63 | 0 | 0 | 0 | 0 |
| Camallanus lacustris | Generalist^9^ | 0.34 | 4 | 0 | 24.93 | 0 | 0 | 0 | 0 |
| Tylodelphis clavata | Generalist^10^ | 0.37 | 25 | 0 | 15.34 | 0 | 0 | 0 | 0 |
| Raphidascaris acus | Generalist^9^ | 0.16 | 3 | 0 | 13.15 | 0.42 | 4 | 4 | 29.14 |
| Contracaecum sp. | Generalist^9^ | 0.09 | 4 | 0 | 7.12 | 0 | 0 | 0 | 0 |
| Paradilepis scolecina | Generalist^11^ | 0.07 | 2 | 0 | 5.48 | 0 | 0 | 0 | 0 |
| Anguillicoloides crassus | Generalist^9^ | 0.05 | 1 | 0 | 4.93 | 0.05 | 2 | 0 | 5.14 |
| Proteocephalus filicollis | Specialist^12^ | 0.05 | 2 | 0 | 4.38 | 0 | 0 | 0 | 0 |
| Phyllodistomum folium | Generalist^6^ | 0.04 | 2 | 0 | 2.74 | 0 | 0 | 0 | 0 |
| Valipora campylancristrota | Generalist^11^ | 0.03 | 4 | 0 | 1.37 | 0 | 0 | 0 | 0 |
| Ichthyophthirius multifiliis | Generalist^3^ | 0.01 | 1 | 0 | 1.10 | 0.02 | 1 | 0 | 1.43 |
| Ergasilius sp. | Generalist^8^ | 0.01 | 1 | 0 | 0.82 | 0 | 0 | 0 | 0 |
| Piscicola | Generalist^6^ | 0.00 | 1 | 0 | 0.27 | 0.01 | 1 | 0 | 0.86 |
| Acanthocephalus lucii | Generalist^13^ | 0 | 0 | 0 | 0 | 0.06 | 2 | 0 | 6 |

**References supplementary table 1.**

^1^Chappell LH, Hardie LJ & Secombes CJ (1994) Diplostomiasis: the disease and host-parasite interactions. In: Parasitic Diseases in Fish (Eds AW Pike & JW Lewis), pp. 59-86. Samara Publishing Limited, Dyfed.

^2^Vojtek J (1964) Zur Kenntnis des Entwicklungszyklus von Apatemon cobitidis (Linstow, 1890). Z f Parasitenkd 24: 578-599

^3^Lom J & Dykova I (1992) Protozoan parasites of fishes. Elsevier, Amsterdam

^4^Blazek R & Gelnar M (2006) Temporal and spatial distribution [Temporal and spatial distribution of glochidial larval stages of European unionid mussels (Mollusca : Unionidae) on host fishes. Folia Parasitologica 53:98-106](http://apps.webofknowledge.com/full_record.do?product=UA&search_mode=GeneralSearch&qid=9&SID=W1bpLa@JmnCkb@aJcpH&page=2&doc=13)

^5^Glaeser HJ (1974) Six new species of the Gyrodactylus-wageneri group monogenea gyrodactylidae with remarks on preparation determination terminology and host specificity Zool. Anzeiger 192: 56-76

^6^[I. E. Bykhovskaya-Pavlovskaya](http://openlibrary.org/authors/OL4329058A/I._E._Bykhovskaya-Pavlovskaya) IE (1964) [Key to parasites of freshwater fish of the U.S.S.R.](http://openlibrary.org/books/OL17903579M/Key_to_parasites_of_freshwater_fish_of_the_U.S.S.R.). Israel Program for Scientific Translations, Jerusalem

^7^Beaver PC (1941) The life history of Echinochasmus *donaldsoni* n. sp., a trematode (Echinostomidae) from the pied-billed grebe. Jour. Parasitol 27: 347-354    DOI: 10.2307/3272817

^8^Hoffman GL (1999) Parasites of North American freshwater fishes, 2. Edn. Comstock Publishing Associates/Cornell University Press, Ithaca and London

^9^Moravec, F. (1994) Parasitic nematodes of freshwater fishes in Europe. Kluwer Academic Publishers, Dordrecht

^10^Kennedy CR (1974) Checklist of British and Irish freshwater fish parasites with notes on their distribution. J Fish Biol 6: 613-644.

^11^[Scholz T](http://apps.webofknowledge.com/OneClickSearch.do?product=UA&search_mode=OneClickSearch&colName=WOS&SID=W1bpLa@JmnCkb@aJcpH&field=AU&value=Scholz, T&ut=14269760&pos={2}) , [Bray RA](http://apps.webofknowledge.com/OneClickSearch.do?product=UA&search_mode=OneClickSearch&colName=WOS&SID=W1bpLa@JmnCkb@aJcpH&field=AU&value=Bray, RA&ut=2159103&pos={2}), [Kuchta R](http://apps.webofknowledge.com/OneClickSearch.do?product=UA&search_mode=OneClickSearch&colName=WOS&SID=W1bpLa@JmnCkb@aJcpH&field=AU&value=Kuchta, R&ut=12331422&pos={2}) & [Repova R](http://apps.webofknowledge.com/OneClickSearch.do?product=UA&search_mode=OneClickSearch&colName=WOS&SID=W1bpLa@JmnCkb@aJcpH&field=AU&value=Repova, R) (2004) Larvae of gryporhynchid cestodes (Cyclophyllidea) from fish: a review. Folia Parasitologica 51: 131-152

^12^Scholz T (1999) Life cycles of species of *Proteocephalus*, parasites of fishes in the palearctic region: a review. J Helminthol 73: 1-19

^13^Brattey J (1988) Life-history and population biology of adult Acanthocephalus lucii (Acanthocephala, Echinorhynchidae). J Parasitol 74: 1
