# Supplementary material for: Divergent selection on locally adapted major histocompatibility complex immune genes experimentally proven in the field
Source: Ecol Lett. 2012 May 15;15(7):723–31. doi: 10.1111/j.1461-0248.2012.01791.x (PMC3440595; doi:10.1111/j.1461-0248.2012.01791.x)
Supplement: Supplementary file 6 [file ele0015-0723-SD6.doc]

**Supplementary Table 2:** Statistical details for the variance components analysis. Shannon index was tested in a linear mixed effect model with habitat of exposure, fish line (Pure vs. hybrids), Family, MHC origin and mesocosm ID. Variance components are derived from (100xVariance)/ sum (Variance) where variance= (standard deviation from linear mixed effect model for each variable)^2^.

| **Variables** | **Std. Dev.** | **Variance explained** |
| --- | --- | --- |
| Habitat of exposure | 0.23 | 37.9% |
| Line (Pure vs. Hybrids) | 0.17 | 20.71% |
| Family | 0.15 | 16.12% |
| MHC Origin | 0.12 | 10.32% |
| Mesocosm ID | 0.12 | 10.32% |
| Residuals | 0.08 | 4.59% |
