# Supplementary material for: Divergent selection on locally adapted major histocompatibility complex immune genes experimentally proven in the field
Source: Ecol Lett. 2012 May 15;15(7):723–31. doi: 10.1111/j.1461-0248.2012.01791.x (PMC3440595; doi:10.1111/j.1461-0248.2012.01791.x)
Supplement: Supplementary file 7 [file ele0015-0723-SD7.doc]

**Supplementary Tables 3:** Statistical details for the linear mixed effect models on a) Shannon parasite index (total parasite load) for all four MHC genotypes, b) Shannon parasite index only for LL and RR genotypes and c) the specialist *Gyrodactylus sp.* (Number of infecting Gyrodactylus parasites) with only LL and RR MHC genotypes. Both Mesocosm ID and family were set as random factors.

| **Table 3.a. Term** | **Estimate** | **St. error** | **d.f. den** | **t ratio** | **Prob>|t|** |
| --- | --- | --- | --- | --- | --- |
| **Intercept** | 0.0018 | 0.0288 | 4.3872 | 0.06 | 0.9520 |
| **Habitat[Lake]** | 0.1661 | 0.0236 | 40.0058 | 7.03 | **<.0001** |
| **Origin[LL]** | -0.0626 | 0.0343 | 573.4787 | -1.82 | 0.0687 |
| **Origin[LR]** | 0.0671 | 0.0364 | 255.3284 | 1.84 | 0.0665 |
| **Origin[RL]** | -0.0414 | 0.0285 | 452.3278 | -1.45 | 0.1468 |
| **Habitat[Lake]*Origin[LL]** | -0.0964 | 0.0197 | 601.5818 | -4.90 | **<.0001** |
| **Habitat[Lake]*Origin[LR]** | 0.0678 | 0.0331 | 619.4779 | 1.75 | 0.0806 |
| **Habitat[Lake]*Origin[RL]** | 0.0140 | 0.0259 | 616.9662 | 0.54 | 0.5866 |
|  |  |  |  |  |  |
| **Table 3.b. Term** | **Estimate** | **St. error** | **d.f. den** | **t ratio** | **Prob>|t|** |
| **Intercept** | -0.0448 | 0.0431 | 8.6690 | -1.04 | 0.3271 |
| **Habitat[Lake]** | 0.1188 | 0.0353 | 25.4824 | 3.37 | **0.0024** |
| **Origin[LL]** | -0.0528 | 0.0255 | 130.3261 | -2.07 | **0.0405** |
| **Habitat[Lake]*Origin[LL]** | -0.0310 | 0.0205 | 133.0245 | -1.22 | 0.2232 |
|  |  |  |  |  |  |
| **Table 3.c. Term** | **Estimate** | **St. error** | **d.f. den** | **t ratio** | **Prob>|t|** |
| **Intercept** | 16.5924 | 4.3160 | 8.6813 | 3.84 | 0.0042 |
| **Habitat[Lake]** | -12.8596 | 3.5186 | 25.4903 | -3.65 | **0.0012** |
| **Origin[LL]** | 5.2966 | 2.5458 | 130.3125 | 2.08 | **0.0394** |
| **Habitat[Lake]*Origin[LL]** | 3.0574 | 2.5285 | 133.0282 | 1.21 | 0.2287 |
