# Supplementary material for: Divergent selection on locally adapted major histocompatibility complex immune genes experimentally proven in the field
Source: Ecol Lett. 2012 May 15;15(7):723–31. doi: 10.1111/j.1461-0248.2012.01791.x (PMC3440595; doi:10.1111/j.1461-0248.2012.01791.x)
Supplement: Supplementary file 8 [file ele0015-0723-SD8.doc]

**Supplementary Tables 4:** a) Summary of the linear mixed effect model conducted on the Shannon parasite index with individual average amino acid p-distance between alleles (corrected for the number of MHC alleles), habitat of exposure and MHC origin as predictors. Mesocosm ID and family were set as random factors. b) Summary of the linear mixed effect model conducted on the fish weight with *Gyrodactylus* load, MHC origin and habitat of exposure as predictors. Weight is expressed as residuals from a linear model with sex as predictor. Mesocosm ID and family were set as random factors.

| **Table 4.a. Term** | | **Estimate** | | **St. error** | | **d.f. den** | | **t ratio** | | **Prob>|t|** |
| --- | --- | --- | --- | --- | --- | --- | --- | --- | --- | --- |
| **Intercept** | | 1.6750 | | 0.2158 | | 256.3805 | | 7.76 | | <.0001 |
| **Origin[LL]** | | -0.0107 | | 0.0594 | | 266.2418 | | -0.18 | | 0.8575 |
| **Origin[LR]** | | 0.0399 | | 0.0441 | | 216.1241 | | 0.90 | | 0.3670 |
| **Origin[RL]** | | -0.0321 | | 0.0338 | | 274.0988 | | -0.95 | | 0.3431 |
| **Habitat[Lake]** | | 0.1415 | | 0.0222 | | 25.6410 | | 6.36 | | **<.0001** |
| **MHC divergence** | | -0.1262 | | 0.0533 | | 271.1289 | | -2.37 | | **0.0185** |
| **Habitat[Lake]*Origin[LL]** | | 0.0102 | | 0.0337 | | 274.9834 | | 0.30 | | 0.7623 |
| **Habitat[Lake]*Origin[LR]** | | -0.0630 | | 0.0338 | | 253.8211 | | -1.87 | | 0.0633 |
| **Habitat[Lake]*Origin[RL]** | | 0.0304 | | 0.0266 | | 273.1040 | | 1.14 | | 0.2542 |
| **MHC divergence*Origin[LL]** | | 1.2160 | | 1.3217 | | 251.1367 | | 0.92 | | 0.3584 |
| **MHC divergence *Origin[LR]** | | -0.0941 | | 0.7327 | | 222.2003 | | -0.13 | | 0.8979 |
| **MHC divergence *Origin[RL]** | | -1.6309 | | 0.6452 | | 264.0396 | | -2.53 | | **0.0121** |
|  |  | |  | |  | |  | |  | |
| **Table 4.b. Term** | **Estimate** | | **St. error** | | **d.f. den** | | **t ratio** | | **Prob>|t|** | |
| **Intercept** | 1907.4351 | | 71.7153 | | 10.3760 | | 26.60 | | <0.001 | |
| **Habitat[Lake]** | -144.0435 | | 34.6445 | | 8.1927 | | -4.16 | | **0.0030** | |
| **Nb of Gyrodactylus sp.** | 0.8868 | | 0.7098 | | 87.3796 | | 1.25 | | 0.2149 | |
| **Origin[LL]** | 16.9860 | | 37.1530 | | 139.9918 | | 0.46 | | 0.6482 | |
| **Origin[LL]*Gyrodactylus** | -1.6738 | | 0.7590 | | 81.2742 | | -2.21 | | **0.0303** | |
